# Semi-automated quantification of left ventricular volumes and ejection fraction by real-time three-dimensional echocardiography

**DOI:** 10.1186/1476-7120-7-18

**Published:** 2009-04-20

**Authors:** Jøger Hansegård, Stig Urheim, Ketil Lunde, Siri Malm, Stein Inge Rabben

**Affiliations:** 1 GE Vingmed Ultrasound, Horten, Norway; 2 Dept. of Cardiology, Rikshospitalet University Hospital, Oslo, Norway; 3 Harstad University Hospital, Harstad, Norway

## Abstract

**Background:**

Recent studies have shown that real-time three-dimensional (3D) echocardiography (RT3DE) gives more accurate and reproducible left ventricular (LV) volume and ejection fraction (EF) measurements than traditional two-dimensional methods. A new semi-automated tool (4DLVQ) for volume measurements in RT3DE has been developed. We sought to evaluate the accuracy and repeatability of this method compared to a 3D echo standard.

**Methods:**

LV end-diastolic volumes (EDV), end-systolic volumes (ESV), and EF measured using 4DLVQ were compared with a commercially available semi-automated analysis tool (TomTec 4D LV-Analysis ver. 2.2) in 35 patients. Repeated measurements were performed to investigate inter- and intra-observer variability.

**Results:**

Average analysis time of the new tool was 141s, significantly shorter than 261s using TomTec (*p *< 0.001). Bland Altman analysis revealed high agreement of measured EDV, ESV, and EF compared to TomTec (*p *= *NS*), with bias and 95% limits of agreement of 2.1 ± 21 ml, -0.88 ± 17 ml, and 1.6 ± 11% for EDV, ESV, and EF respectively. Intra-observer variability of 4DLVQ vs. TomTec was 7.5 ± 6.2 ml vs. 7.7 ± 7.3 ml for EDV, 5.5 ± 5.6 ml vs. 5.0 ± 5.9 ml for ESV, and 3.0 ± 2.7% vs. 2.1 ± 2.0% for EF (*p *= *NS*). The inter-observer variability of 4DLVQ vs. TomTec was 9.0 ± 5.9 ml vs. 17 ± 6.3 ml for EDV (*p *< 0.05), 5.0 ± 3.6 ml vs. 12 ± 7.7 ml for ESV (*p *< 0.05), and 2.7 ± 2.8% vs. 3.0 ± 2.1% for EF (*p *= *NS*).

**Conclusion:**

In conclusion, the new analysis tool gives rapid and reproducible measurements of LV volumes and EF, with good agreement compared to another RT3DE volume quantification tool.

## Background

Left ventricular (LV) volumes and ejection fraction (EF) are important parameters for diagnosis and prognosis of patients with heart disease [1-3]. Traditionally, LV volumes are measured by manual tracing in two sequentially acquired two-dimensional (2D) echocardiograms, using the biplane method of disks (MOD) [4,5]. The spatial under-sampling of the ventricle, inherent with such 2D techniques, requires geometric assumptions about the LV shape. Foreshortening, occurring when the image plane is oblique to the ventricular main axis, also introduces errors in MOD measurements in 2D echocardiography [6,7].

Real-time three-dimensional (3D) echocardiography (RT3DE) (also known as four-dimensional (4D) echocardiography) is gaining popularity as a routine clinical tool [8], and has a significant potential of improving clinical decision-making [9]. Of particular interest is the improved accuracy and repeatability of volume and EF measurements, compared to conventional 2D techniques [6,10-13]. However, manual analysis of 3D data is time-consuming and impractical. Thus, clinical use of volume measurements from RT3DE requires simple and efficient automated analysis tools.

Currently, two commercially available volume measurement tools for RT3DE exist on the market; QLAB (Philips, Andover, Massachussetts, USA), and TomTec 4D LV-Analysis (TomTec Imaging Systems, Unterschleissheim, Germany). Different versions of the TomTec tool have been verified against cardiac magnetic resonance imaging (cMRI) in several studies [7,14-16], showing excellent agreement of measured LV volumes and EF. One of the challenges with the TomTec analysis tool is that it requires manual tracing of the endocardial border in three apical planes for initialization and manual correction of the detected surface [17]. Manual tracing of the endocardial boundary is a difficult and time-consuming procedure, especially for non-expert users, and the accuracy is experience dependent [12].

GE has introduced a new semi-automated tool for 4D LV volume quantification (4DLVQ) in RT3DE (EchoPAC ver. 108.1.0, GE Vingmed Ultrasound, Horten, Norway). 4DLVQ provides a simple user interface and an efficient workflow by eliminating the need for manual tracing, making the tool simple to use for non-expert users.

The objective of this study was to evaluate the agreement of LV volumes and EF measured by 4DLVQ compared to TomTec, to evaluate the repeatability of these parameters, and to determine the potential of 4DLVQ as a clinical tool.

## Methods

### Volume quantification tool

4DLVQ is a volume quantification tool for rapid semi-automated detection of the LV endocardial border in RT3DE. When entering the tool, the user is presented with a quad-screen, showing cine loops of three apical views with 60° inter-plane spacing, and one short axis (SAX) view. If required, the apical views can be manually corrected to show the standard apical four-chamber (A4CH), apical two-chamber (A2CH), and apical long axis (ALAX) views, thereby eliminating foreshortening. When this anatomical alignment step is complete, the ED frame is automatically detected from the EGG, but can be manually corrected if necessary.

**Figure 1:**
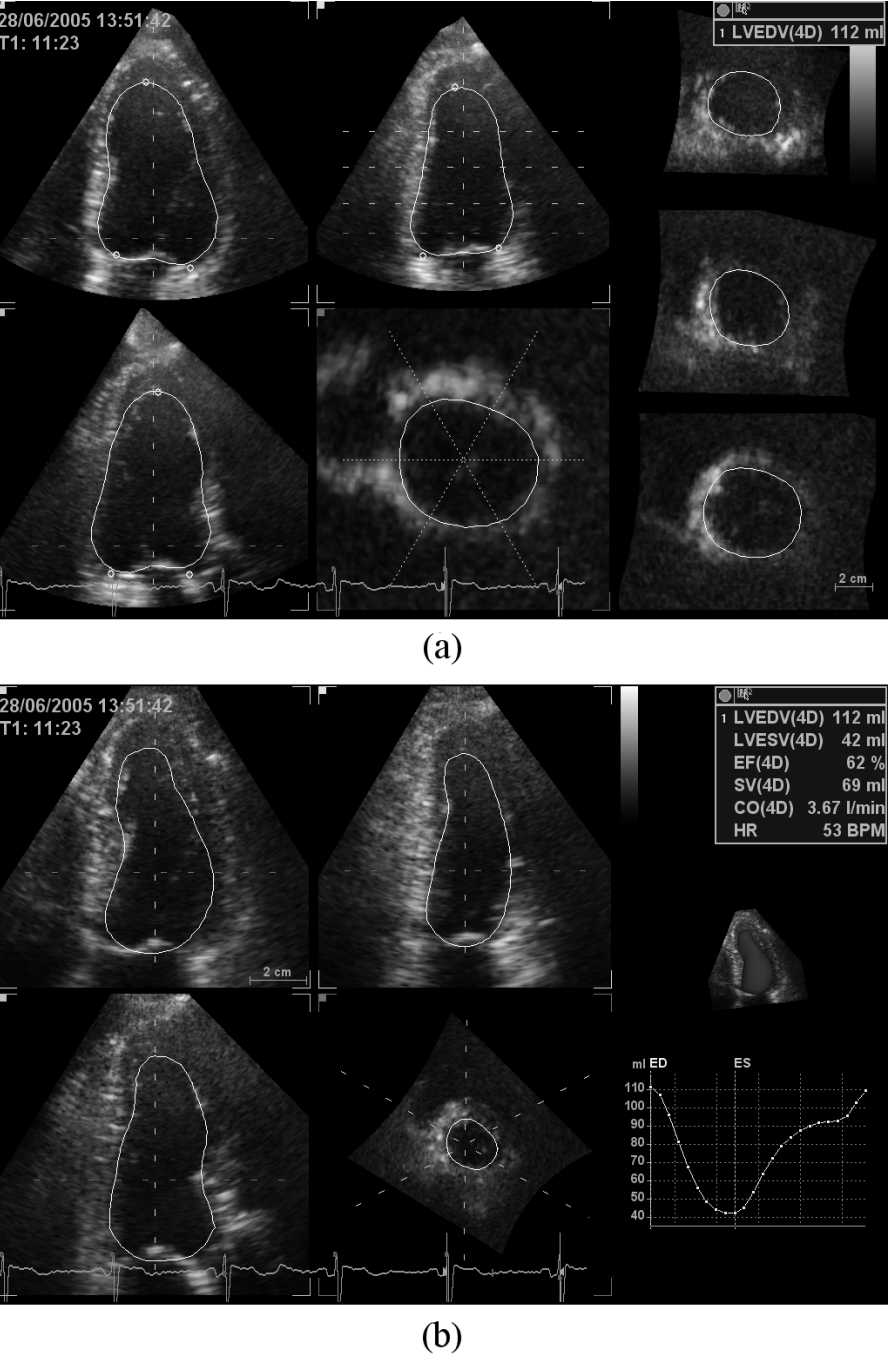
**An example of LV surface detection at ED using 4DLVQ is shown in (a)**. The standard apical views (top left, top middle, bottom left) were obtained by manual alignment. Each small circle in the apical views indicates a manually defined landmark used for initialization of surface detection. One SAX view (middle, bottom) was dynamically updated to reflect the trackball position, in order to facilitate precise landmark positioning and verification of the 3D surface detection. If necessary, additional landmarks could be added to leave papillary muscles within measured cavity volume. Three extra SAX views distributed between apex and base (right) were used to further verify the detected surface. The complete 4D surface detection at ES with time-volume curve is shown in (b).

**Figure 2:**
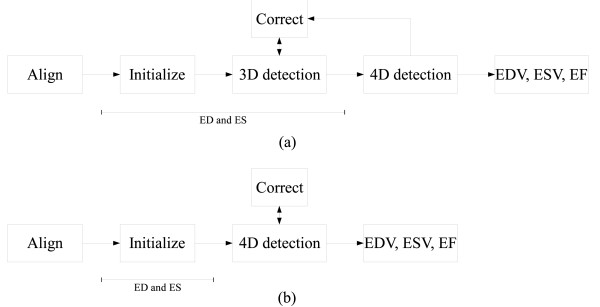
**Comparison of workflow for 4DLVQ (a) and TomTec (b)**. With 4DLVQ, each view is aligned to the standard apical view. Initialization is done at ED using 3 clicks in each apical view. 3D surface detection is automatically triggered, and if necessary the detected 3D surface is edited manually by adding landmarks. This is an interactive procedure where the 3D surface detection is repeated after each new landmark. Once completed with ED, the same procedure is repeated for ES. When both ED and ES are finished, a full 4D surface detection is performed to obtain EDV, ESV, and EF along with a time-volume curve. At this stage it is possible to edit the surfaces if improvements are required. With TomTec, initialization is done by accurate tracing at both ED and ES in each view after alignment to the standard apical views. 4D surface detection is done directly after initialization, and correction is done to the resulting surfaces if required.

While displaying the ED frame, surface detection is initialized by manually selecting two points identifying the mitral annulus and one point identifying the LV apex in each of the three apical views shown in figure 1(a). After the total of nine landmarks are defined at ED, non-temporal 3D surface detection is immediately performed to extract the endocardial border and to compute the EDV. The time required for a full 3D surface detection is less than one second. Cross-sections of the detected 3D surface are displayed in three apical views and three SAX views distributed between the LV apex and base, as shown in figure 1(a), to allow visual verification of the detected surface. A fourth user controlled SAX plane is used to further inspect the surface detection result. If necessary, the displayed LV surface can, at this time, be edited manually by adding or removing landmark points. An interactive workflow is achieved by automatically repeating 3D surface detection after each new landmark until a satisfactory result is obtained. An overview of the tool workflow is given in figure 2(a).

The above procedure is repeated for ES. The frame corresponding best to end systole is estimated automatically from the R-R interval of the EGG [18,19] and can be corrected manually.

When surface detection is complete for both ED and ES, preliminary measurements of EDV, ESV, and EF are presented to the user. However, better measurements can be achieved by manually triggering temporal 4D surface detection to detect LV surfaces for each frame in the entire cardiac cycle. This typically requires less than 10 seconds, depending on the frame- and heart rate, and gives a full time-volume curve (see additional file 1 and additional file 2). Once completed, the maximum and minimum volumes are presented on the screen as EDV and ESV respectively along with the derived EF, as shown in figure 1(b). If required, the LV surfaces computed during this step can be edited further. In these cases, non-temporal 3D surface detection is performed after each additional manually defined landmark as for ED and ES. A full 4D surface detection can then be manually triggered once again, to update the time-volume curve. Several segmentation algorithms suitable for 3D echocardiography exist [20]. Commonly, these algorithms are formulated as deformable surface models, such as for instance level sets [21,22], or simplex meshes [23,24].

4DLVQ is based upon a 3D energy minimizing deformable model [25]. The deformable model is evolved using an iterative deformation scheme, under the influence of internal and image derived forces, temporal forces, and user defined landmark forces.

The model's internal forces ensure second order shape continuity of the detected object by counteracting stretching and bending of the surface. In order to be able to adapt the deformable model to clinical data, where a large variation of LV shapes is expected due to different pathologies and individual variations between patients, the deformable model is allowed to evolve freely, and is not constrained by any explicit a priori shape model.

The segmentation algorithm is initialized at ED and ES by manually selecting nine initial points in three different image planes rotated about the LV long axis. These manually defined initial points are used to position a simplified geometric LV model within the LV cavity. The radius and height of the initial model are determined from the position of the manually defined initial points.

Image forces are derived from the volumetric data using a local edge detector, utilizing a combination of gradient and transition information [26]. These image forces pull the surface towards image edges within a region around the deformable model. To ensure consistent surface detection from frame to frame, and to give a smooth time-volume curve, temporal forces constrain the model to second-order temporal continuity.

By disabling temporal forces, the deformable model can be used for surface detection in single frames. This mode was used for initial surface detection at end-diastole (ED) and end-systole (ES).

User input is facilitated by generating spring-like forces that pull the deformable model towards the spatial location of user-defined landmarks [27].

The relative weights of the image and model forces were tuned on more than 100 training data sets to obtain a good trade-off between accuracy and robustness in different imaging situations and for various pathologies.

After completed surface detection, LV volumes were derived from the triangulated surfaces by summation of all triangular patches using the divergence theorem [28].

### Patient selection

Evaluation was done in 3D echocardiograms recorded in conjunction with two previous studies, giving data from a total of 56 patients (77% men, age 23–76) referred for echocardiography at St. Olavs Hospital, Trondheim, Norway, ([29], *n *= 20, exclusion criteria: atrial fibrillation, severe non-cardiac diseases), and Rikshospitalet University Hospital, Oslo, Norway, ([30], *n *= 36, exclusion criterion: arrhythmia). Of note is that the original studies did not use 3D data. 3D echocardiograms were therefore only recorded in randomized sub-groups of the total study populations, as an addition to the primary protocols.

Participants were not pre-selected from 2D or 3D image quality for the primary protocol, nor for the sub-group subject to 3D echocardiography. All patients gave informed written consent to participation, and the studies conformed to the declaration of Helsinki, with approval from the regional committees of medical ethics.

Apical volumetric imaging was performed by experienced operators using a Vivid 7 scanner (GE Vingmed Ultrasound, Horten, Norway) and a 3D transducer (3V). Electrocardiogram (EGG) gated sub-volumes were acquired at 20–30 frames per second from four consecutive cardiac cycles during breath-hold, with the participants in the left lateral recumbent position. The sub-volumes were automatically stitched to a sequence of full 3D volumes covering the entire LV, and stored digitally for analysis.

In 20 (36%) of the 56 patients, less than 70% of the myocardium was visualized, caused by a too narrow imaging sector, shadows, or dropouts. Stitching artefacts were found in one additional patient (2%). These factors would prohibit reliable volume measurements [31], and the total of 21 patients (38%), were therefore excluded from further analysis.

### Analysis

A customized EchoPAC workstation (GE Vingmed Ultrasound, Horten, Norway) with the 4DLVQ software integrated was used by an expert operator according to the described tool workflow. Accurate anatomical alignment to the standard A4CH, A2CH, and ALAX views, were applied in all cases. If necessary, the ED frame was corrected to agree with the maximum cavity area, while the ES frame was adjusted primarily to agree with aortic valve closing, secondarily by visually determining the minimal cavity area. We always applied 4D segmentation to compute the full time-volume curve, as our experience was that this gave the most accurate EDV and ESV estimates. Manual editing of the detected surfaces was done as necessary at ED, ES, and after performing 4D segmentation. When a satisfactory result was obtained, EDV, ESV, and EF, were recorded for analysis.

Repeated measurements were done in a randomized order using an EchoPAC workstation with a TomTec 4D LV-Analysis plug-in ver. 2.2. by the same expert operator after a minimum of 14 days. The operator was equally experienced with both 4DLVQ and TomTec, and was blinded to previous measurements. An overview of the TomTec workflow is given in figure 2(b). The apical views were aligned to the standard A4CH, A2CH, and ALAX views, similarly as for the 4DLVQ tool, to eliminate foreshortening.

Initialization of the TomTec surface detection algorithm was done by manually tracing the endocardial border using a spline-based annotation tool in the three apical views. Contrary to 4DLVQ, manual tracing was completed at both ED and ES in each apical view before continuing to the next view. Manual editing of the LV surface after surface detection could be avoided by tracing the endocardial border as accurately as possible during initialization [17]. While tracing, a cine-loop of the corresponding view displayed the traced endocardial border to ensure temporal consistency between ED and ES. After tracing in the A4CH, A2CH, and the ALAX views, 4D LV surface detection was performed. Two manually adjustable apical planes were used to validate the automatically detected surfaces. The tool provided a manual editing feature for the detected LV surfaces, but since the initialization procedure required the user to accurately trace the LV according to his preference, this was not used. EDV, ESV, and EF measurements were derived from the automatically detected LV surfaces, and recorded for analysis. These values were used as reference values for evaluation of 4DLVQ.

For both methods, the analysis time was measured from the start of analysis of volumetric data until the volume and EF measurements were displayed on the screen. The analysis time was reported as average time ± standard deviation (SD).

### Repeatability

Intra-observer variability for 4DLVQ and TomTec was assessed in all 35 patients by the primary operator in a randomized order. The second expert operator assessed inter-observer variability in 10 randomly selected patients. Both operators were equally experienced with both 4DLVQ and TomTec, and were blinded to previous measurements. The minimum time interval between repeated measurements was 14 days.

To compare repeatability with other studies, we adopted the method of Jacobs et al. [11] and Sugeng et al. [13]. First, the absolute difference between two repeated measurements was computed for each patient as shown in Eq. (1). The absolute differences were reported as mean ± SD over all patients. Secondly, relative percentage variability was defined as the absolute difference between two single measurements, normalized by the average of the two measurements in the same patient as shown in Eq. (2). The relative percentage variability was reported as mean ± SD over all patients.

(1)

(2)

### Statistical analysis

The relationship between 4DLVQ and the TomTec reference values was analyzed by linear regression and Bland Altman analysis [32]. The latter was used to evaluate the agreement between the two methods (two-tailed t-test on the differences with a null hypothesis of zero difference and *p *< 0.05 regarded as significant). The agreement between the two methods was reported as the mean difference (bias) and the corresponding 95% limits of agreement. For comparison with other studies, Pearson's correlation coefficient between the two methods was also reported.

Agreement between inter- and intra-observer variability of the two methods was computed from the absolute differences between repeated measurements (Mann-Whitney U test with *p *< 0.05 regarded as a significant).

Analysis times were compared using a Mann-Whitney U test with *p *< 0.05 regarded as significant.

## Results

Semi-automated analysis was feasible in all 35 data sets. A full 4D analysis using 4DLVQ required 141 ± 37 s including image alignment, initialization, and manual correction of the detected surfaces. This was significantly quicker than for Tom Tec (*p *< 0.001), which required 261 ± 63 s. The maximum time required for the two tools was 266 and 392 seconds respectively. The 4DLVQ tool required on average three additional manually defined landmarks to correct the initially detected surfaces.

**Figure 3:**
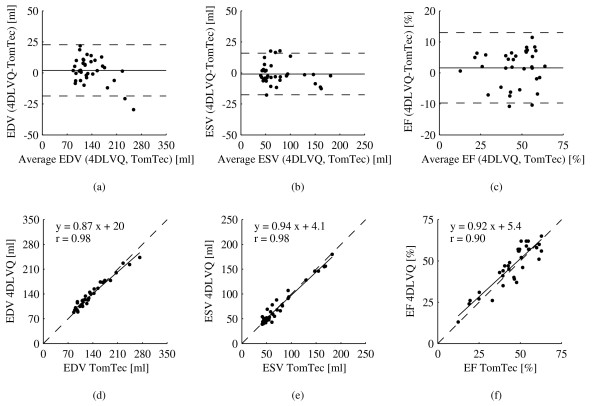
**Bland Altman analysis of EDV, ESV, and EF measured by 4DLVQ compared to TomTec is shown in (a), (b), and (c) respectively**. Average difference (solid) is shown along with 95% limits of agreement (dashed). EDV, ESV, and EF measured by 4DLVQ are plotted against TomTec in (d), (e), and (f) along with unit line (dashed), regression line (solid), and Pearson's correlation coefficient r.

TomTec and 4DLVQ yielded similar results for population mean and range for EDV, ESV, and EF (Table 1). Figure 3(a–c) shows the agreement between 4DLVQ and TomTec. The mean differences and 95% limits of agreement for EDV were 2.1 ± 21 ml, -0.88 ± 17 ml for ESV, and 1.6 ± 11% for EF. The differences between the two tools were not statistically significant for EDV, ESV, or EF. The small bias and relatively narrow 95% limits of agreements indicate that 4DLVQ gives results that compare well with TomTec. Figure 3(d–f) shows the relationship between EDV, ESV, and EF measured by 4DLVQ and TomTec. Linear regression gave slopes that were less than unity for all parameters (0.87, 0.94, and 0.92 for EDV, ESV, and EF respectively). The zero intercepts were 20 ml for EDV, 4.1 ml for ESV, and 5.4% for EF. The correlation coefficients of parameters measured by 4DLVQ and TomTec were 0.98, 0.98, and 0.90 for EDV, ESV, and EF respectively.

**Table 1:** Population mean and range of EDV, ESV, and EF measured by 4DLVQ and TomTec.

	EDV [ml]	ESV [ml]	EF [%]
4DLVQ	137 (88–243)	76 (39–180)	47 (13–65)
TomTec	135 (86–273)	77 (41–182)	45 (12–63)

The results from the inter- and intra-observer analysis of 4DLVQ and TomTec are shown in Table 2. This analysis reveals low intra-observer variability for both methods. An important observation is that the inter-observer variability was significantly better with 4DLVQ than with TomTec for both EDV and ESV.

**Table 2:** Inter- and intra-observer variability for TomTec and 4DLVQ.

	Inter-observer		Intra-observer	
	Abs.	%	Abs.	%

EDV				
4DLVQ [ml]	9.0 ± 5.9	5.9 ± 3.7	7.5 ± 6.2	5.8 ± 4.5
TomTec [ml]	17* ± 6.3	11 ± 4.5	7.7 ± 7.3	5.5 ± 5.2
ESV				
4DLVQ [ml]	5.0 ± 3.6	5.6 ± 4.0	5.5 ± 5.6	8.0 ± 7.6
TomTec [ml]	12* ± 7.7	15 ± 8.8	5.0 ± 5.9	6.0 ± 7.3
EF				
4DLVQ [%]	2.7 ± 2. 8	5.9 ± 5.8	3.0 ± 2.7	7.1 ± 6.5
TomTec [%]	3.0 ± 2.1	8.5 ± 6.5	2.1 ± 2.0	4.9 ± 4.4

* Significantly different from 4DLVQ (*p *< 0.05). EDV, end-diastolic volume; ESV, end-systolic volume; EF, ejection fraction. Abs. values are population mean ± SD of absolute differences between repeated measurements. % values are population mean ± SD of absolute differences of repeated measurements normalized by the average of the two repeated measurements.

## Discussion

We have evaluated a new semi-automated method for rapid quantification of LV volumes and EF in volumetric echocardiograms, and compared this to a reference volume quantification tool (TomTec) in 35 patients.

3D echocardiography makes it possible to capture the shape and function of the entire LV in a single data set. Compared to 2D echocardiography, this is an advantage for LV quantification, since geometric assumptions of LV shape can be completely eliminated. Further, 3D echocardiograms allowed for manually aligning the displayed views to the true anatomical LV main axis to avoid foreshortening and to ensure precise identification of the LV apex. These factors make automated 3D quantification better suited for accurate and reproducible measurements of ventricular volumes and EF than manual 2D methods. Also, an automated method can provide time-volume curves from a full cardiac cycle, giving more accurate EF estimates. The time-volume curve can potentially also be used to improve echocardiographic diagnosis, by providing information about timing of cardiac events, and filling rates in diastolic function analysis.

Analysis time was significantly shorter with 4DLVQ than with TomTec. This difference was mainly due to the simplified 4DLVQ initialization procedure, requiring only nine easily located landmarks at ED and ES, instead of triplane tracing used for the TomTec analysis. Because of this simplified initialization, a few additional manually defined landmarks were often required to include papillary muscles in the LV volume, but since this was done interactively with immediate visual feedback, the overhead was minimal. One might argue that the TomTec analysis time could be reduced by a less accurate initialization, but it has been shown that accurate initialization is required to avoid time-consuming manual editing of the detected LV surface when using TomTec [17]. Soliman et al. [16] reported a TomTec analysis time of 360 ± 120 seconds, confirming that our results are representable with respect to TomTec analysis time.

We have shown that EDV, ESV, and EF assessed by 4DLVQ compare well to measurements performed by TomTec, with small bias, narrow 95% limits of agreement, and high repeatability. A limitation of this study is the lack of an independent reference, and it is therefore not possible to determine which of the two methods is more accurate. However, since the agreement between the two methods was high for EDV, ESV, and EF, we conclude that 4DLVQ performs at least as well as TomTec in clinically realistic data, even with a lower analysis time.

Several studies have presented repeatability assessment for various versions of TomTec. Soliman et al. [16] reported inter-observer variability of 6.4 ± 7.8 ml, 7.8 ± 9.7 ml, and 7.1 ± 6.9%, and intra-observer variability of 4.7 ± 3.2 ml, 6.1 ± 5.8 ml, and 6.6 ± 7.4% for EDV, ESV, and EF respectively. Our results demonstrated similar intra-observer variability, but higher inter-observer variability with TomTec. We speculate if the discrepancy in inter-observer variability can be explained by the manual initialization procedure provided by TomTec. Slight differences in tracing conventions caused a bias between the observers of approximately 9 ml at ED and ES. This bias corresponds to a systematic tracing error of less than 1 mm for typical chamber sizes [12], but the impact on the variability parameters used in this study is evident. 4DLVQ requires less user input during initialization, and is therefore less influenced by such differences in tracing conventions, as confirmed by a lower inter-observer variability with 4DLVQ than with TomTec.

Differences between the workflows provided by TomTec and 4DLVQ are illustrated in figure 2. With 4DLVQ, initialization of all views is completed at ED before continuing to ES. TomTec uses a different strategy, where manual initialization is completed at both ED and ES in each view before proceeding with the next view. The traced contours are shown in a cine-loop preview display during tracing, providing additional information to ensure consistent contours between ED and ES. It has been claimed that manual editing in TomTec only has local impact on the detected surfaces [16]. We experienced in several cases that manually editing the surface caused it to "slip" from the endocardial border outside of the edited area, even in cases with strong edge evidence. Also, TomTec does not provide immediately updated surface detection during editing, whereas 4DLVQ provided immediate feedback, giving better control over the detected surface.

The clinical feasibility of RT3DE relies on simple and efficient analysis tools, ideally integrated as a part of the scanner software to facilitate on-line analysis during examinations. This puts strong constraints on the performance and ease of use of the tool, also with respect to manual correction of automatically detected surfaces. The presented volume quantification tool was implemented as an off-line analysis tool for use on the EchoPAC workstation. We have shown that a full 4D analysis can be done in less than 3 minutes, also in patients where manual correction was needed. This indicates that the tool is well suited for on-line analysis.

4DLVQ seems to be a reliable clinical tool, which provides rapid and reproducible measurements of LV volumes and EF with good agreement compared to TomTec. It has a simple workflow that makes it easy to use for non-expert users. Recent development within the field of automated landmark detection in RT3DE [33] may be utilized to completely eliminate the need for manual initialization. Also, promising results have been presented using fully automated real-time 4D surface detection methods [34,35]. In patients with poor acoustic properties, semi-automated methods are still preferred, since they allow for manual correction of the automatically determined LV volumes. But in the future, fully automated methods will improve efficiency and repeatability of echocardiography examinations.

It has been shown that the accuracy of LV volumes and EF is highly correlated to the amount of myocardium that is visualized in the RT3DE recording [31]. We defined an image quality threshold of 70% myocardium visibility, causing exclusion of 36% of the patients (which were not pre-selected for image quality). The need for combining sub-volumes from four cardiac cycles caused exclusion of one patient due to inability to hold breath throughout the acquisition. Future improvements to probe design and front-end processing capabilities are expected to give increased field of view, and less need for EGG gating, while improving image contrast and signal to noise ratio. These factors will improve feasibility and accuracy of automated assessment of LV function.

In this study, both TomTec and 4DLVQ were evaluated on the same data sets, to rule out differences in measured volumes and EF caused by differences during acquisition related to frame rate, probe position, and differences in image quality. This study design also ruled out comparison with Philips QLAB, due to file format limitations. Analysis of inter-examination variability, and comparison with analysis packages from multiple vendors should be addressed in a future study.

Cardiac MRI is currently accepted as the gold standard for LV quantification, and several studies have shown that TomTec compares well with cMRI, giving good agreement in measured EF, but with slightly under estimated volumes [7,12,14-16]. This bias is explained by differences in how the two modalities visualize trabeculae and valves, and also partial volume effects in cMRI [36]. Since 4DLVQ provides volume and EF measurements that agree well with TomTec, it is reasonable to believe that 4DLVQ will give similar results in a comparison with cMRI. The next natural step is therefore to compare 4DLVQ against cMRI. Accurate EF measurements are of high clinical importance for diagnosis, prognosis, and treatment planning of patients with cardiac diseases. 4DLVQ is now fully integrated as an online measurement tool on the Vivid E9 ultrasound scanners (GE Vingmed Ultrasound, Horten, Norway). This allows for fast and reliable bedside measurements of global LV function without limitations related to foreshortening and geometric modelling of the LV shape.

## Conclusion

We have evaluated a new volume quantification tool for automated EDV, ESV, and EF measurements in volumetric echocardiograms. The tool compared well to a commercially available analysis tool (TomTec 4D LV-Analysis), with higher repeatability, and a significantly shorter analysis time. This is an important step towards wide spread use of RT3DE in clinical routine.

## Competing interests

JH worked as consultant for GE Vingmed Ultrasound. SIR is employed by GE Vingmed Ultrasound. SU, KL, and SM received research grants from GE Vingmed Ultrasound.

## Authors' contributions

JH contributed to development of the analysis tool, was the primary observer, drafted the manuscript, and performed the statistical analysis. SIR designed and developed the analysis tool, was the secondary observer, supervised the project, and participated in drafting the manuscript. SU collected data, supervised the clinical aspects of the project, and participated in drafting the manuscript. KL collected parts of the data, and revised the manuscript critically for important intellectual content. SM collected parts of the data, and revised the manuscript critically for important intellectual content. All authors read and approved the final manuscript.

## Supplementary Material

Additional file 1 **Temporal 4D (3D + time) analysis results from a normal left ventricle with normal function**. The movie shows four anatomically aligned cut planes through a 4D recording of a normal left ventricle, where left ventricular (LV) surfaces (green contours) have been detected for each frame in the entire cardiac cycle. Manually added landmark points are shown as green circles. The time-volume curve (right) shows the volumes computed from the detected surfaces in each frame of the cardiac cycle, along with the end diastolic (ED) and end systolic (ES) frames computed from the maximum and minimum volumes respectively. Click here for file

Additional file 2 **Temporal 4D (3D + time) analysis results from a dilated left ventricle**. The movie shows four anatomically aligned cut planes through a 4D recording of a dilated left ventricle, where left ventricular (LV) surfaces (green contours) have been detected for each frame in the entire cardiac cycle. Manually added landmark points are shown as green circles. The time-volume curve (right) shows the volumes computed from the detected surfaces in each frame of the cardiac cycle, along with the end diastolic (ED) and end systolic (ES) frames computed from the maximum and minimum volumes respectively. Click here for file
